# Study on the Adsorption Deformation of a Substrate via Spin Coating Based on the 3D-DIC Method and Its Effect on the Homogeneity of Perovskite Films

**DOI:** 10.3390/ma16155454

**Published:** 2023-08-03

**Authors:** Chunhua Ren, Zhishun Zhou, Shuming Cao, Mengting Jiao, Dongyang Xue

**Affiliations:** School of Mechanical Engineering, Tianjin University of Commerce, Tianjin 300134, China

**Keywords:** spin coating, float glass, digital image correlation method, strain, hyperspectral

## Abstract

The physical and chemical stability of perovskite films has always been a key issue for their industrialization, which has been extensively studied in terms of materials, environment, and encapsulation. Spin coating is one of the most commonly used methods for the preparation of perovskite films in research. However, little attention has been paid to the deformation state of the substrate when it is fixed by means of adsorption and its impact. In this work, the three-dimensional digital image correlation (3D-DIC) method and hyperspectral technology are used to acquire and analyze the adsorption deformation characteristics of the substrate during spin coating, as well as the resulting inhomogeneity. Plastic and four different thicknesses of float glass (0.2, 0.5, 0.7, 1.1 mm) were selected as substrates, and they were tested separately on two suction cups with different structures. The results show that the plastic and 0.2 mm specimens exhibit obvious strain localization behavior. The distribution and magnitude of the strain are affected by the size of the sucker structure, especially the width of the groove. For glass specimens, this effect shows a nonlinear decrease with increasing substrate thickness. Compared to the strain value, the irregularity of local deformation has a greater impact on the non-uniform distribution of materials. Finally, inhomogeneities in the perovskite films were observed through optical lens and hyperspectral data. Obviously, the deformation of the substrate caused by adsorption should attract the attention of researchers, especially for flexible or rigid substrates with low thickness. This may affect the centrifugal diffusion path of the precursor, causing microstructure inhomogeneity and residual stress, etc.

## 1. Introduction

As a new generation of photoelectric materials with great potential, perovskite has excellent photoelectric properties, such as a high light absorption coefficient, an adjustable band gap and high carrier mobility. Meanwhile, it also has the advantages of a simple preparation process [1], low cost [2] and flexibility [3]. However, the physical and chemical stability of perovskite thin films in humid heat [4,5,6], ultraviolet light [7] and other environments has always been a key issue preventing their industrialization.

As we all know, the spin-coating method is the earliest and most commonly used method to prepare perovskite solar cells [8,9,10,11]. A large amount of experimental research work in the current literature is based on this approach. Its basic principle is that the substrate is adsorbed on the suction cup using a vacuum pump, and then the perovskite precursor liquid is evenly dispersed on the surface of the substrate via the high-speed rotation of the suction cup. Finally, the nanometer-thick perovskite layer is formed. However, although many perovskite devices used in the study of physical and chemical properties are prepared based on spin-coating, there are few studies that consider and evaluate the influence of adsorption on substrate deformation. In fact, the local deformation of the substrate may be caused by the adsorption and release process, which will inevitably affect the crystallization uniformity and interfacial stress state of the perovskite film. Studies have shown that the quality and crystallinity of the film have a direct impact on the photoelectric conversion efficiency (PCE) and chemical stability of perovskite solar cells [12,13,14]. Simultaneously, the internal stress state of the film is also closely related to the photoelectric properties and long-term stability [15,16]. In addition, tensile and compressive strains have opposite effects on chemical degradation [17]. An in-depth study of the effect of the adsorption process on different substrates is of great significance for comprehensively revealing the formation of micro-defects, the origin and distribution of residual stress, and the mechanical stability of perovskite films.

In recent years, the digital image correlation (DIC) method, a non-contact optical measurement technique, has gained significant popularity in various engineering fields [18,19], including aerospace, materials, and biology. This method offers several advantages [20], such as high measurement accuracy, simplicity of operation, and multi-scale capabilities. For instance, Pan et al. measured the thermal expansion coefficient of thin films using DIC [21]. Emanuela et al. utilized this technology to identify and analyze defects in glass structures [22]. Dai et al. investigate the deformation process of the inner sidewalls using 3D-DIC [23]. Mehrabian et al. quantitatively measured the out-of-plane displacement of composites [24]. Therefore, DIC technology can be effectively employed to study and analyze substrate deformation characteristics induced by adsorption.

In this paper, we investigated the three-dimensional deformation characteristics of different substrates under adsorption using 3D-DIC. Additionally, we employed microscopic hyperspectral technology to observe the inhomogeneity present in perovskite films.

## 2. Materials and Methods

### 2.1. Specimen Preparation

**Figure 1:**
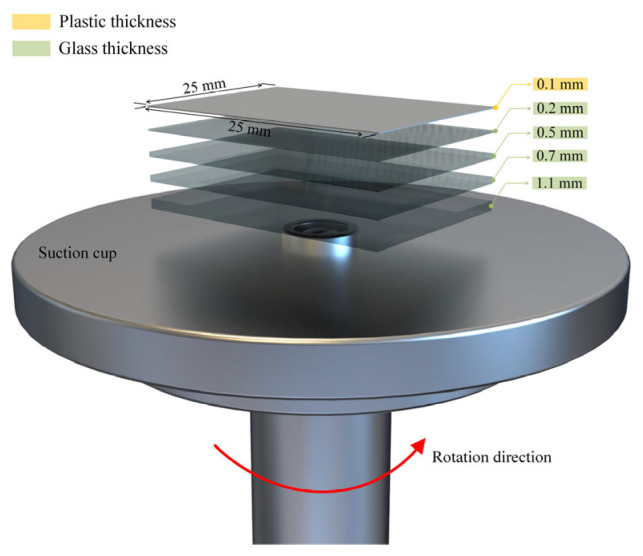
Schematic diagram of a suction cup and different substrates.

Referring to the data report on the thickness of conductive glass substrate in perovskite devices [25,26,27,28], this study selected four different thicknesses of float glass without a conductive layer as the research objects: 0.2 mm, 0.5 mm, 0.7 mm, and 1.1 mm. Additionally, to emphasize the role of adsorption force, a plastic substrate capable of producing flexible perovskite films was used as the control group, with a thickness of 0.1 mm. The specimen size was 25 mm × 25 mm, as depicted in Figure 1. Prior to the experiment, the specimen was successively immersed in absolute ethanol (96–99% conc.) and deionized water for 15 min of ultrasonic cleaning, respectively. It was then dried and treated with ultraviolet-ozone (UV-O_3_) for 15 min to obtain a clean surface with a certain level of adhesion.

### 2.2. Deformation Tests Procedures

The 3D-DIC method was used to measure the full-field strains and out-of-plane displacements of the regions of interest (ROI). The principle of this optical measurement technique is using random patterns on the specimen surface as information carriers [29], and capturing the displacement by tracking the grayscale variations within subsets of the undeformed and deformed images [30,31]. In terms of computation, it involves the cross-correlation function for evaluating the matching degree between the reference subsets and the target subsets [19], as well as sub-pixel search algorithms [32]. In this study, the 3D-DIC system and software used were the VIC-SNAP and Vic-3D v7.2.6 model from Correlated Solutions Inc. (CSI), Irmo, SC, USA.

**Figure 2:**
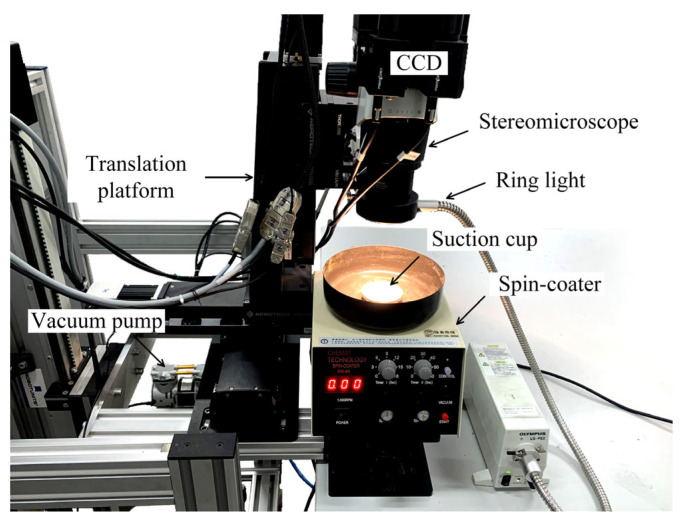
Three-dimensional deformation measurement system for adsorbed specimens.

The deformation image acquisition system under adsorption state is depicted in Figure 2. The system comprised a stereo microscope, two charge-coupled devices (CCDs), a ring light source, a displacement platform, and a spin coater (KW-4A, Chemat Technology, Northridge, CA, USA) equipped with a vacuum pump (power: 40 W). To thoroughly investigate the impact of adsorption force on substrate deformation, two suction cups with different diameters were selected for observation, and the diameters *d* were 10 mm and 25.5 mm, respectively. During the experiment, the glass specimen was placed without any constraints, flatly positioned at the center of the suction cup of the spin-coater. A ring light source was utilized to provide uniform illumination. The focal length and aperture were adjusted to achieve a clear image of the specimen surface. The obtained grayscale image had a size of 2448 × 2048 pixels, with a spatial size of 4.2 μm for each pixel.

**Figure 3:**
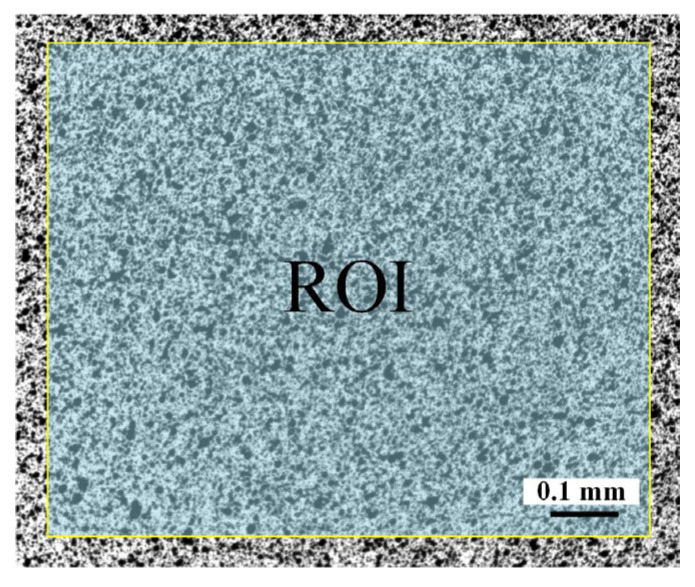
Region of interest for 3D-DIC calculation.

In regard to the speckle pattern, a dense and uniform white base layer was first sprayed onto the specimen surface, followed by the preparation of small black speckle dots using ink and a spray gun. Before the deformation test, the accuracy of the measurement system was evaluated through a zero-deformation experiment. During the experiment, the first step was to capture the specimen image before adsorption, which served as the reference image for DIC calculations. Then, the vacuum pump was activated, and after a stable adsorption period of 10 s, the corresponding deformation images were acquired. Finally, the VIC-3D software was used for deformation measurement after adsorption. Figure 3 illustrates the ROI, with a size of 2240 pixels × 1880 pixels. The sub-region for calculation was set to 35 pixels × 35 pixels, with a step size of 5 pixels.

### 2.3. Microscopic Hyperspectral Testing

**Figure 4:**
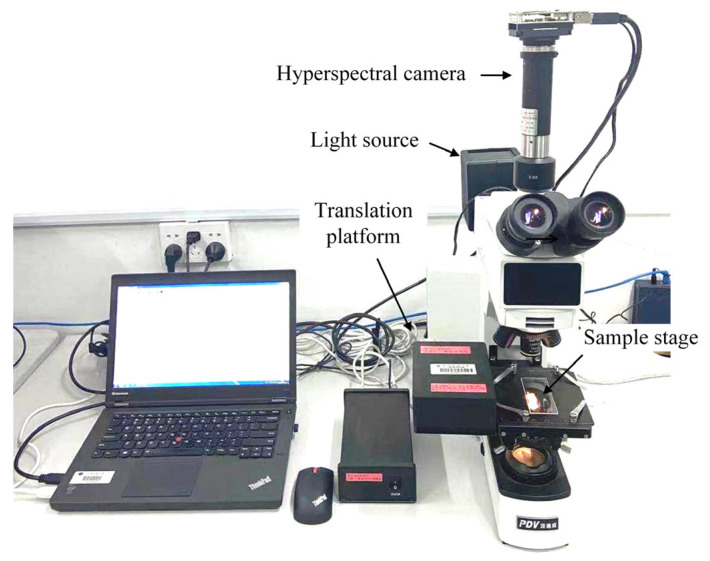
Microscopic hyperspectral test system.

Hyperspectral technology has been used to study the inhomogeneous characteristics of coatings [33,34,35], primarily due to the differences in the spectral responses of films with different thicknesses. In this study, the organic–inorganic hybrid CH_3_NH_3_PbI_3_ perovskite film was prepared by means of spin-coating using the 0.2 mm glass substrate. To evaluate the impact of adsorption on film uniformity, the microscopic hyperspectral instrument with a reflection mode was used to obtain spectral data from the center, groove, and other regions of the film. The testing system is depicted in Figure 4. The main components include an optical microscope, a spectral camera, a translation platform, and a light source. This system is capable of measuring wavelengths ranging from 400 to 900 nm, with a spectral resolution of 2.8 nm. Due to variations in the intensity of the light source across different wavelengths and the presence of dark current noise in the sensor, a black–white calibration was conducted before testing. This calibration process yielded a relative spectral image (*I_r_*) that helped to mitigate interference.

(1) $$I_{r}=\frac{I_{s}-I_{d}}{I_{w}-I_{d}}$$

where *I_s_* is the original hyperspectral image, *I_d_* is the dark field calibration image, and *I_w_* is the whiteboard calibration image.

## 3. Results and Discussion

### 3.1. Reliability of Test System

The zero-deformation experiment was used to evaluate the reliability of the measurement system. A series of seven consecutive images of the unadsorbed state were captured and labeled sequentially as 1–7. Taking the first image as the reference, DIC was used to calculate the other images. The calculation parameters were kept consistent with the adsorption deformation experiment. Table 1 shows the average values and standard deviations of the displacement field measured in the zero-deformation experiment. Here, *u* is the horizontal displacement, *v* represents the vertical displacement, and *w* is the out-of-plane displacement. It can be observed that, although the displacement in the unadsorbed state should be zero, there is some fluctuation in the results. In fact, this is mainly caused by random errors arising from factors such as the experimental system and testing environment. The maximum values for the mean and standard deviation are 0.962 μm and 0.815 μm, respectively, and they both exist in the displacement along the *z*-axis. Obviously, the measurement error for out-of-plane displacement is slightly larger.

### 3.2. The Substrate Deformation under a 10 mm Suction Cup Structure

**Figure 5:**
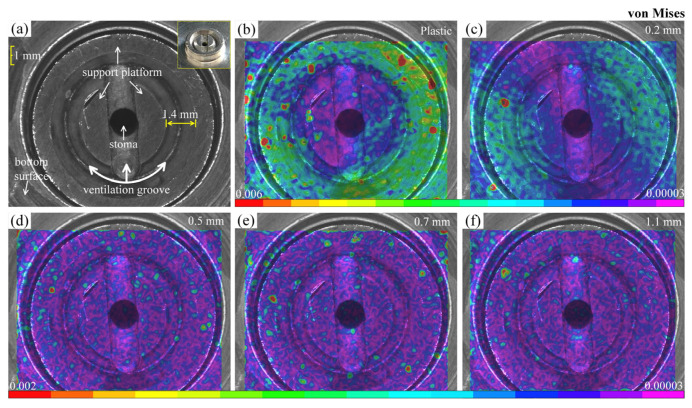
von Mises strain fields of different substrates using the 10 mm-diameter suction cup: (**a**) central structure of the suction cup; (**b**) plastic substrate; (**c**–**f**) glass substrates with different thicknesses.

**Figure 6:**
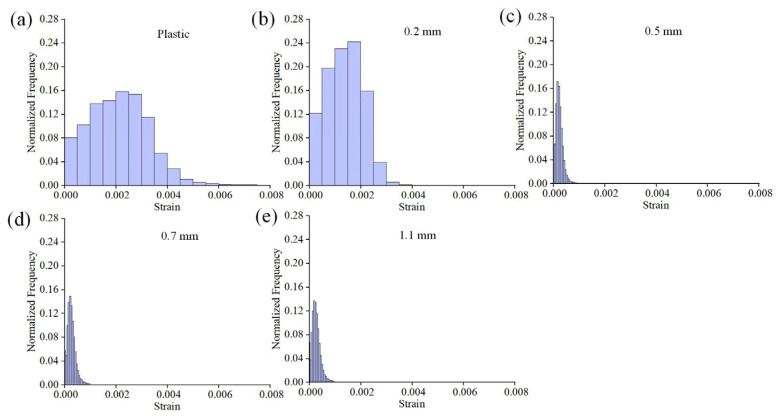
Histograms of the normalized von Mises strain distributions for the data shown in Figure 5: (**a**) plastic substrate; (**b**–**e**) glass substrates with different thicknesses.

The relative deformation could be characterized using von Mises equivalent strain *ε_VM_*. For surface deformation, the ε*_VM_* is expressed as [36]:

(2) $$\varepsilon_{VM}=\sqrt{{\varepsilon_{xx}}^{2}+{\varepsilon_{yy}}^{2}-\varepsilon_{xx}\varepsilon_{yy}+3{\varepsilon_{xy}}^{2}}$$

where *ε_xx_* and *ε_yy_* are the values of the normal strain along the *x* and *y* axes, respectively, while *ε_xy_* is the shear strain components. Figure 5a displays the schematic diagram of the main components of the 10 mm diameter suction cup position, including the support platform, ventilation groove, and stoma. Figure 5b–f shows von Mises strain fields of plastic and glass substrates with different thicknesses in the adsorbed state. It can be observed that the strain field in the plastic substrate (Figure 5b) exhibits a significant non-uniform distribution. Larger deformations are primarily concentrated on the annular support platform, while the deformations near the stoma are smaller. From a structural standpoint, the fixation of the substrate by adsorption is primarily due to the continuous evacuation of gas from the annular grooves by the vacuum pump. As a result, the substrate areas corresponding to the grooves experience downward forces, leading to displacement away from the surface. However, this displacement is prevented by the two intermediate semicircular support platforms, resulting in minimal deformation. Meanwhile, the outer annular support platform, acting as the constrained end, will cause significant stress concentration. Regarding glass substrates, the 0.2 mm-thick specimen (Figure 5c) also shows an obvious strain localization phenomenon, and presents a certain symmetry. This is primarily due to the high rigidity of glass, which makes it difficult to undergo continuous deformation corresponding to the position of adsorption. The deformation fields of 0.5 mm and other thickness specimens are relatively random and uniform, which indicates that the adsorption force has little influence on the substrate surface. The histograms illustrate the normalized von Mises strain distributions, as shown in Figure 6. It is evident that the strain is higher in the plastic and 0.2 mm-thick specimens. The plastic substrate displays a broader distribution and non-uniformity. In contrast, the strain in specimens with a thickness of 0.5 mm or more is noticeably reduced, and they exhibit a similar trend. This could be attributed to the potential influence of measurement errors.

**Figure 7:**
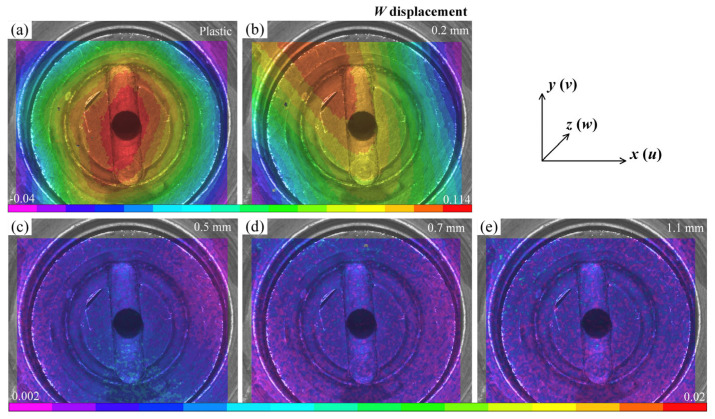
Off-plane displacement *w* of different substrates using the 10 mm diameter suction cup: (**a**) plastic substrate; (**b**–**e**) glass substrates with different thicknesses.

**Figure 8:**
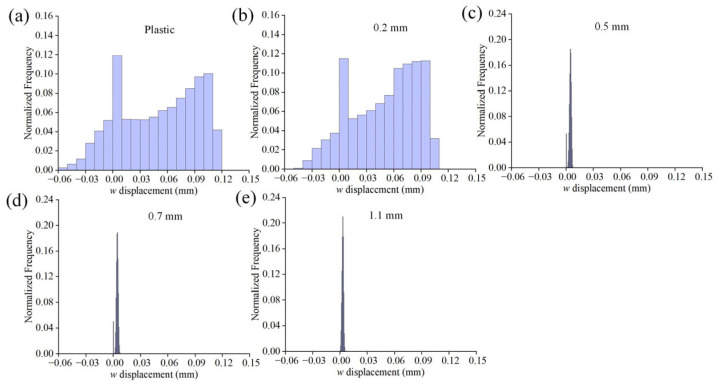
Histograms of the normalized *w* distributions for the data shown in Figure 7: (**a**) plastic substrate; (**b**–**e**) glass substrates with different thicknesses.

Figure 7 illustrates the *w* displacement field for different substrates. It is evident that there are distinct gradients of the out-of-plane displacement in the plastic and 0.2 mm glass specimens (Figure 7a,b). The maximum displacement occurs at the stoma, while the junction of the groove and support platform displays a layered feature. For the 0.2 mm glass specimen, the distribution of *w* presents the inhomogeneous characteristics of a fan-shaped topography, whose orientation is close to the vertical grooves. In contrast, the *w* fields in other thicknesses are more dispersed, without any significant impact. Figure 8 displays the histograms of the normalized *w* distributions for each specimen. In the plastic and 0.2 mm specimens, it can be observed that as the displacement increases, the frequency also gradually rises. This suggests that a substantial portion of the regions experience notable out-of-plane displacements and are characterized by a non-uniform distribution state. Those for the specimens with other thicknesses are considerably smaller. Combining the characteristics of the deformation field, this indicates that despite the higher stiffness of float glass [37], the adsorption effect of the coater can also induce localized deformation behavior. This effect shows a nonlinear decrease with increasing substrate thickness.

### 3.3. The Substrate Deformation under a 25.5 mm Suction Cup Structure

**Figure 9:**
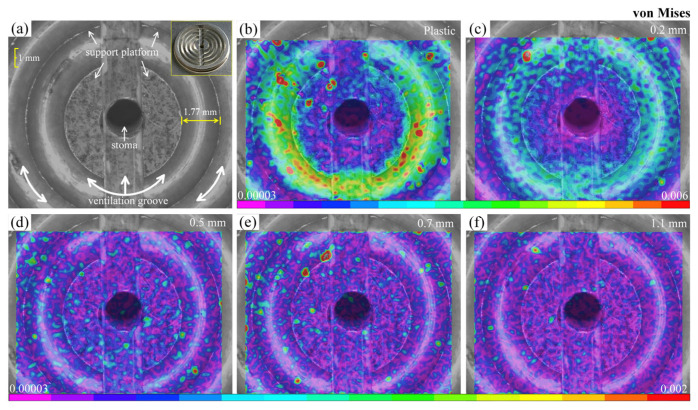
von Mises strain fields of different substrates using the 25.5 mm-diameter suction cup: (**a**) central structure of the suction cup; (**b**) plastic substrate; (**c**–**f**) glass substrates with different thicknesses.

**Figure 10:**
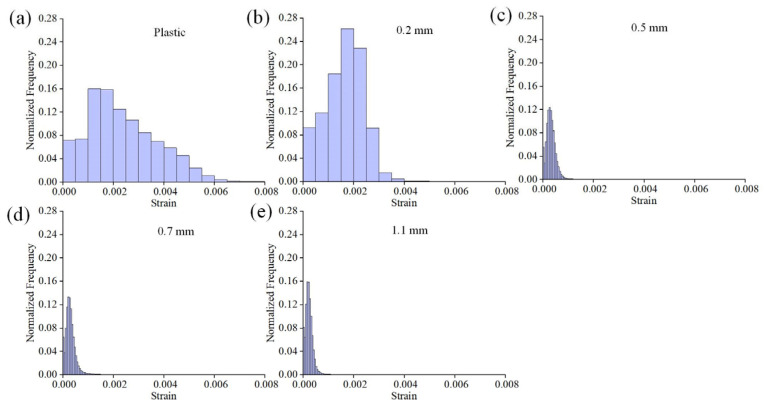
Histograms of the normalized von Mises strain distributions for the data shown in Figure 9: (**a**) plastic substrate; (**b**–**e**) glass substrates with different thicknesses.

Figure 9a shows the structure at the center of the suction cup with the diameter of 25.5 mm. Due to the increased diameter, only the stoma, some support platforms, and ventilation grooves at the central position can be observed under the same magnification. Similar to the calculated results for the 10 mm-diameter suction cup, there is also a noticeable localized deformation phenomenon in both the plastic and 0.2 mm glass substrates (Figure 9b,c). The strain distribution exhibits a circular pattern, which corresponds well with the structure of the suction cup. The areas with larger strains are mainly located above the annular grooves, indicating that they are caused by the suction force of the vacuum pump. In the plastic substrate, there is a larger strain gradient between the grooves and the support platform. However, this is not as pronounced in the 0.2 mm glass specimen. This is because glass has a higher stiffness, resulting in more uniform deformation. Meanwhile, the strain concentration exhibits a block-like dispersed distribution. The deformation field of specimens in other thicknesses is relatively diffuse, and there is no obvious strain concentration. The histogram of the normalized von Mises strain distribution is shown in Figure 10. It can be seen that the peak in the plastic specimen exhibits a pronounced leftward skew, indicating the presence of localized strain concentration. Additionally, the peak on the 0.2 mm substrate is prominent, exceeding 24%. This is primarily attributed to the deformation in the annular groove area. As for the specimens from a 0.5 mm thickness onwards, the deformation is minimal, but there is a slight leftward skew in the peak, likely influenced by system and environmental interference.

**Figure 11:**
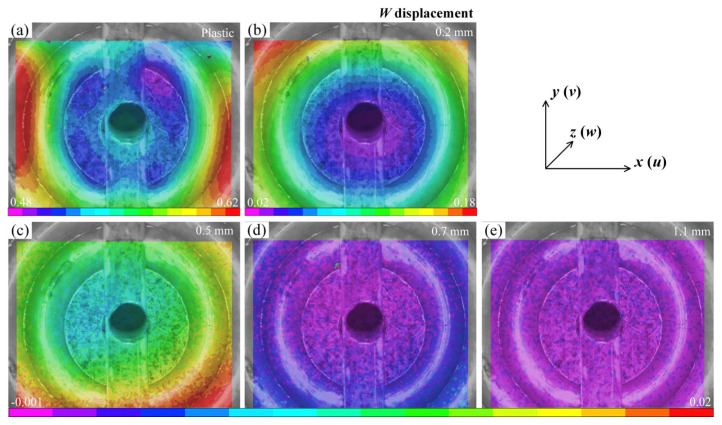
Off-plane displacement *w* of different substrates using the 25.5 mm diameter suction cup: (**a**) plastic substrate; (**b**–**e**) glass substrates with different thicknesses.

**Figure 12:**
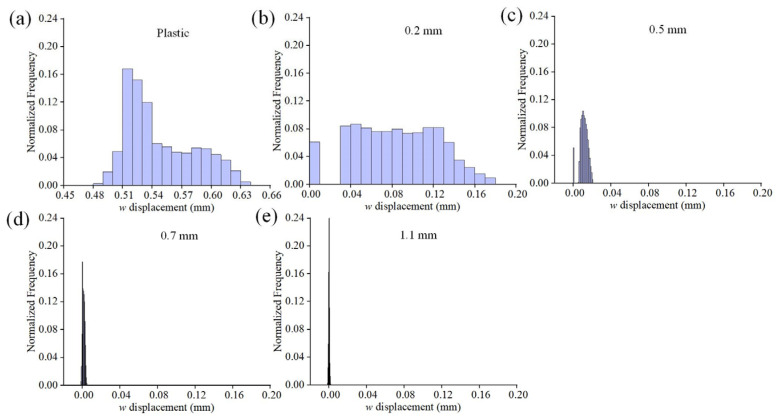
Histograms of the normalized *w* distributions for the data shown in Figure 11: (**a**) plastic substrate; (**b**–**e**) glass substrates with different thicknesses.

Figure 11 shows the *w* displacement field of different substrates under the 22.5 mm diameter suction cup. It can be observed that as the specimens thickness increases, the displacement away from the surface transitions from a localized concentration to a more diffuse distribution. For the plastic and thinner specimens (Figure 11a–c), the *w* mostly increases gradually from the center to the edge, which is opposite to the change trend of the 10 mm suction cup. Meanwhile, there is a gradient change at the junction of the support platform and the groove. From the histogram of the plastic substrate (Figure 12a), it can be observed that its peak is shifted to the left and exhibits a significant density gradient. Furthermore, compared to the 10 mm suction cup, the out-of-plane displacement has increased several times. This upward trend is also evident in the 0.2 mm and 0.5 mm substrate (Figure 12b,c). This may be related to the size of the ring groove. The width of the 25.5 mm suction cup is about 1.77 mm, which is about 26.4% larger. From a mechanical perspective, under the same load conditions, a greater distance between the points of constraint will result in a larger bending moment [38]. Obviously, although the large-size suction cup is more stable in the process of carrying the substrate, its groove structure and size may have a greater impact on the adsorption deformation. Therefore, optimizing the geometry and size of the suction cup is a way to reduce the adsorption effect.

### 3.4. Inhomogeneity Analysis of the Perovskite Films

**Figure 13:**
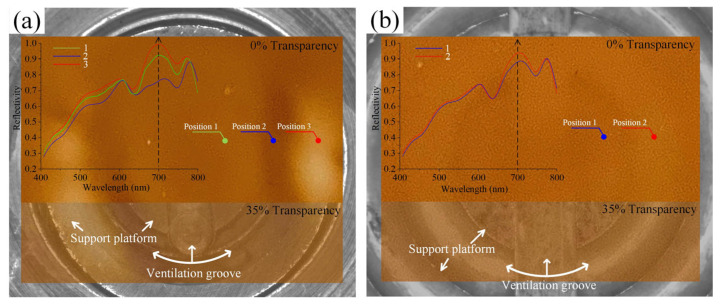
Optical images and the average spectra of the same-sized area acquired at different positions: (**a**) 10 mm suction cup; (**b**) 25.5 mm suction cup.

Perovskite films were prepared via spin-coating on the 0.2 mm glass substrate using 10 mm and 25.5 mm suction cups, respectively. The optical images of the thin film were captured and proportionally mapped onto the suction cup structure used for deformation measurements, maintaining positional correspondence. To better illustrate the relationship between the inhomogeneous morphology and the suction cup structure, the film images were separately segmented with 0% and 35% transparency (Figure 13). For the 10 mm suction cup, it clearly exhibits three gradients. The darker colors mainly correspond to the ventilation groove positions, while the lighter colors are located at the support platform. This non-uniform morphology is not continuous and is primarily distributed on both sides of the vertical groove. In comparison, the differences in the 25.5 mm suction cup are relatively small. The lighter colors correspond well to the annular grooves and exhibit a certain degree of continuity. In fact, the obtained deformation field also shows similar distribution characteristics, whether it is von Mises strain or *w* field. To provide a more visual and quantitative representation of the differences, the average spectra of the same size area were acquired from typical positions, as indicated by the labels in Figure 13. By comparing the reflectance data corresponding to the wavelength near 700 nm, it is found that the inhomogeneity of the 10 mm suction cup is more severe, with a maximum difference of 20.4%. It is about 6.4% in the sample of 25.5 mm suction cup. Interestingly, the strain is actually larger when using the 25.5 mm suction cup. This may indicate that the influence caused by the irregularity of substrate deformation is more significant, leading to a more pronounced interference in the centrifugal diffusion path of the precursor liquid. Additionally, even after the adsorption force disappears, the formed but not yet dried perovskite film is still influenced by substrate deformation. Therefore, for flexible or thin rigid substrates, the local deformation caused by adsorption force cannot be ignored. This may reduce the quality of film formation, introduce residual stress, and become one of the important factors affecting the physical and chemical properties of the film [39,40].

## 4. Conclusions

In this study, based on the 3D-DIC method and hyperspectral technology, the deformation laws of plastic and glass substrates with different thicknesses during spin coating were obtained and analyzed, and the spectral response characteristics of perovskite films were explored.

(1) In the 10 mm and 25.5 mm-diameter suction cups, both plastic and 0.2 mm glass showed obvious strain localization behavior during adsorption. The strain field distribution corresponds clearly to the support platform and groove positions.
(2) Compared to the magnitude of strain, the irregularity of localized deformation has a greater impact on the uniformity of the film. This will directly affect the path of centrifugal diffusion of the solution, causing non-uniform distribution of materials.
(3) The inhomogeneity of the perovskite film in the 0.2 mm glass substrate can be seen macroscopically. From the reflectance spectra, the position of the support platform and the groove has a large difference at a wavelength of around 700 nm. The maximum difference is about 20.4%. The local deformation caused by the adsorption force may reduce the quality of film formation and introduce residual stress, especially for flexible or rigid substrates with small thickness.

## Figures and Tables

**Table 1:** The mean values and standard deviation of measured displacement fields in the zero-deformation experiment (Unite: ×10^−3^ mm).

Image Number	2 to 1	3 to 1	4 to 1	5 to 1	6 to 1	7 to 1
Mean values of *u*	0.012	0.312	0.091	0.62	0.313	0.212
Standard deviation of *u*	0.042	0.045	0.054	0.041	0.043	0.041
Mean values of *v*	0.223	0.094	0.331	0.004	0.126	0.106
Standard deviation of *v*	0.004	0.005	0.006	0.005	0.005	0.006
Mean values of *w*	0.344	0.404	0.425	0.962	0.831	0.771
Standard deviation of *w*	0.216	0.353	0.632	0.712	0.815	0.524

## Data Availability

Data are contained within the article.
